# Carrier localization in In-rich InGaN/GaN multiple quantum wells for green light-emitting diodes

**DOI:** 10.1038/srep09373

**Published:** 2015-03-20

**Authors:** Hyun Jeong, Hyeon Jun Jeong, Hye Min Oh, Chang-Hee Hong, Eun-Kyung Suh, Gilles Lerondel, Mun Seok Jeong

**Affiliations:** 1 Center for Integrated Nanostructure Physics (CINAP), Institute for Basic Science (IBS), Sungkyunkwan University, Suwon 440-746, Republic of Korea; 2 Laboratoire de Nanotechnologie et d'Instrumentation Optique, Institut Charles Delaunay, CNRS-UMR 6281, Université de Technologie de Troyes, BP 2060, 10010 Troyes, France; 3 Department of Energy Science, Sungkyunkwan University, Suwon 440-746, Republic of Korea; 4 School of Semiconductor and Chemical Engineering, Chonbuk National University, Jeonju 561-756, Republic of Korea

## Abstract

Carrier localization phenomena in indium-rich InGaN/GaN multiple quantum wells (MQWs) grown on sapphire and GaN substrates were investigated. Temperature-dependent photoluminescence (PL) spectroscopy, ultraviolet near-field scanning optical microscopy (NSOM), and confocal time-resolved PL (TRPL) spectroscopy were employed to verify the correlation between carrier localization and crystal quality. From the spatially resolved PL measurements, we observed that the distribution and shape of luminescent clusters, which were known as an outcome of the carrier localization, are strongly affected by the crystalline quality. Spectroscopic analysis of the NSOM signal shows that carrier localization of MQWs with low crystalline quality is different from that of MQWs with high crystalline quality. This interrelation between carrier localization and crystal quality is well supported by confocal TRPL results.

GaN-based light-emitting diodes (LEDs) have attracted much attention for solid-state lighting applications owing to a strongly reduced energy consumption compared to traditional incandescent light bulbs^1^,^2^,^3^,^4^,^5^,^6^,^7^. Particularly, InGaN active layers in light emitting devices have great scientific interest for the realization of full-color displays due to the tunability of the energy gap along the entire visible range^8^,^9^,^10^,^11^,^12^,^13^,^14^. Owing to these revolutionary benefits, the Nobel Prize in Physics was awarded for InGaN-based blue LEDs in 2014. Typically, InGaN-based quantum wells (QWs) with low In contents (<20%; from near-UV to blue wavelengths) grown on c-plane sapphire substrates have a high internal quantum efficiency (IQE), although GaN crystals contain a high dislocation density (~10^9^ cm^−2^), which is induced by the large lattice mismatch between GaN and sapphire^15^,^16^,^17^,^18^,^19^,^20^,^21^. The high IQE of InGaN-based QWs is attributed to carrier localization phenomena occurring in dot-like In-rich InGaN formed in the InGaN layer^22^,^23^,^24^,^25^. Carrier localization reduces the effect of nonradiative recombination at the dislocations^26^. The dot-like In-rich InGaN clusters are formed by In aggregation and phase separation of the InGaN layer because of the immiscibility of GaN and InN^27^,^28^,^29^. However, the IQE of InGaN-based QWs, which has a spectral range that extends from blue to green wavelengths, drops dramatically because of a crystalline quality issue known as “green gap”^30^,^31^. According to previous reports, the “green gap” has been explained by a high dislocation density that results from a large lattice mismatch between InGaN and GaN. These dislocations lead to an increased nonradiative recombination rate and charge separation that arises from the increased piezoelectric polarization in the QW, leading to a reduced electron-hole wave function overlap^32^,^33^,^34^. To overcome the “green gap”, a number of research groups have investigated the optical properties of InGaN-based QWs. Kaneta et al. suggested that the radiative recombination center of a green LED entirely depends on the local crystalline quality^35^. Deet al. reported that a strong polarization field could arise from an In-rich InGaN region^36^. Despite these reports, clear experimental evidence of the correlation between carrier localization and crystalline quality is still needed. In this paper, we report on a study of In-rich InGaN/GaN multiple quantum wells (MQWs) by using temperature dependent PL spectroscopy, near-field scanning optical microscopy, and confocal time-resolved PL microscopy. A clear evidence of the correlation between the carrier localization and the crystalline quality is reported.

## Results

**Figure 1:**
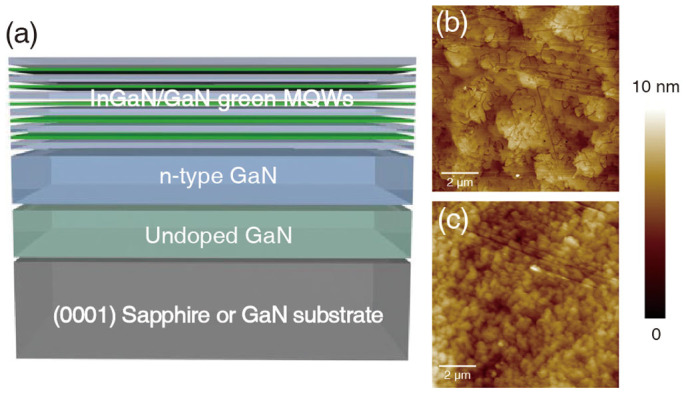
(a) Schematics of In-rich InGaN/GaN MQWs on c-plane sapphire and GaN substrates. The targeted emission wavelength was 520 nm. Topographic AFM images of MQWs on (b) sapphire and (c) GaN. Various crystal defects are revealed by the topography of the MQWs on sapphire.

In this study, in order to compare carrier localization phenomena as a function of the crystalline quality of the epilayers, two different kinds of substrates were used. The first one is a sapphire substrate, which is typically used in the LED industry. In the case of GaN grown on sapphire substrates, the GaN crystal has a high dislocation density of ~10^9^ cm^−2^ due to the large differences of lattice constants and thermal expansion coefficients between sapphire and GaN^37^,^38^. The second is a GaN substrate that is used for the homoepitaxial growth of GaN epilayers. According to previous reports, GaN grown on freestanding GaN substrates possesses a low dislocation density of 10^6^ cm^−2^ owing to a better homogeneity of the grown GaN layer^39^,^40^,^41^. Fig. 1(a) is a schematic view of the sample structure. Un-doped GaN was grown on sapphire and GaN substrates as a buffer layer. To keep the conditions identical to a conventional LED structure, n-type GaN that acts as an electron injection layer was grown on the un-doped GaN layer. Finally, five pairs of InGaN/GaN MQWs emitting at a wavelength of 520 nm were grown on the n-type GaN layer. To define the crystalline quality of both samples, AFM surface scanning was conducted. Before scanning the samples, wet chemical etching was carried out by dipping the samples in diluted HCl (37%) for 1 min to reveal crystallographic defects. Surface topography images of green MQWs on sapphire and GaN templates are shown in Figs. 1(b) and (c), respectively. Various kinds of defects are clearly observed for MQWs located on sapphire, such as edge dislocations and grain boundaries (Fig. 1(b)). On the other hand, only small grain boundaries are observed in MQWs grown on GaN as shown in Fig. 1(c). Surface topography images indicate that the MQWs grown on GaN have a lower crystal defect density than the MQWs sample grown on sapphire.

**Figure 2:**
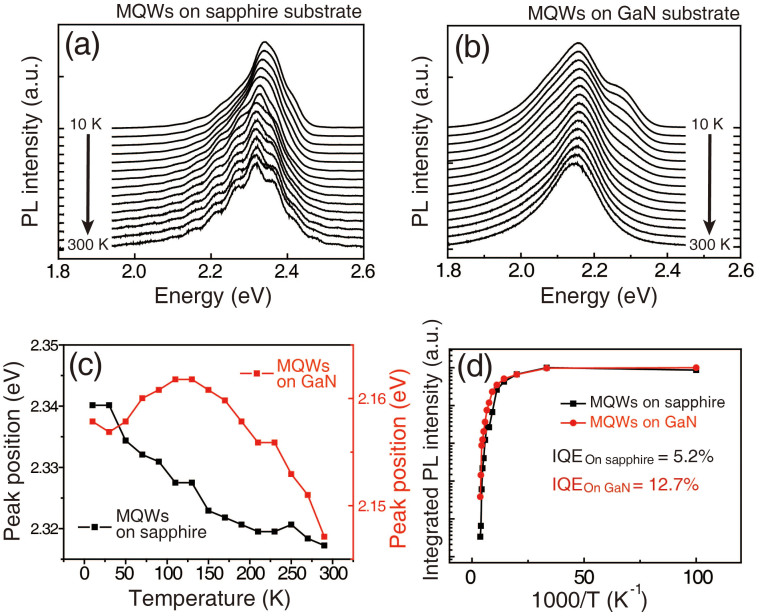
Temperature-dependent PL spectra of MQWs grown on (a) sapphire and (b) GaN substrates. The peak positions of the room-temperature PL spectra for MQWs grown on sapphire and GaN substrates are around 2.3 eV and 2.1 eV, respectively. (c) Changes of the PL spectral peak positions as a function of the temperature. The “S-shaped” variation of the peak position is observed only for MQWs grown on GaN. (d) Arrhenius plots of both samples to estimate the IQE. The IQE of the MQWs grown on sapphire and GaN are 5.2% and 12.7%, respectively.

To probe the carrier localization phenomena for both samples, we first measured the PL spectra as a function of the temperature in a range of 10 K to 300 K. Figs. 2(a) and (b) show temperature-dependent PL spectra of MQWs on sapphire and GaN, respectively. All PL spectra were normalized to compare the peak positions and their shape. The main peak positions for MQWs on sapphire and GaN are located around 2.3 eV and 2.1 eV, respectively. This difference in peak position is attributed to variations of In composition and crystal strain in InGaN QWs, which are induced by the lattice mismatch between the GaN-based structure and the substrate. Fig. 2(c) shows the PL peak position as a function of the temperature for both samples. As the temperature increases, both samples exhibit a red-shift in the peak position. In general, the shrinkage of the energy band gap of both GaN and InGaN epilayers that is observed for increasing temperatures is explained by the Varshni empirical equation, E_g_(T) = E_0_ − αT^2^/(β + T), in which E_0_ is the transition energy at 0 K, and α and β are the Varshni thermal coefficients^42^,^43^. Contrary to the MQWs on the GaN substrate, MQWs fabricated on sapphire did follow the typical temperature dependence of the energy gap shrinkage. On the opposite, a blue-shift of the PL peak position was observed in the temperature range of 30 K to 150 K. This “S-shaped” variation of the peak position as a function of the temperature has been reported to be evidence of carrier localization phenomena in InGaN epitaxial layers^25^. To estimate the IQE of both samples, the Arrhenius plot of the normalized integrated PL intensity for both samples was plotted as shown in Fig. 2(d). Assuming that the IQE at 10 K is 100%, the IQE of MQWs grown on sapphire and GaN are 5.2% and 12.7%, respectively. This higher IQE implies that the density of radiative recombination centers in the MQWs on the GaN sample is higher because of the higher crystalline quality^44^,^45^,^46^. From the temperature-dependent PL spectra, we can conclude that carrier localization phenomena are affected by the crystalline quality of InGaN QWs.

**Figure 3:**
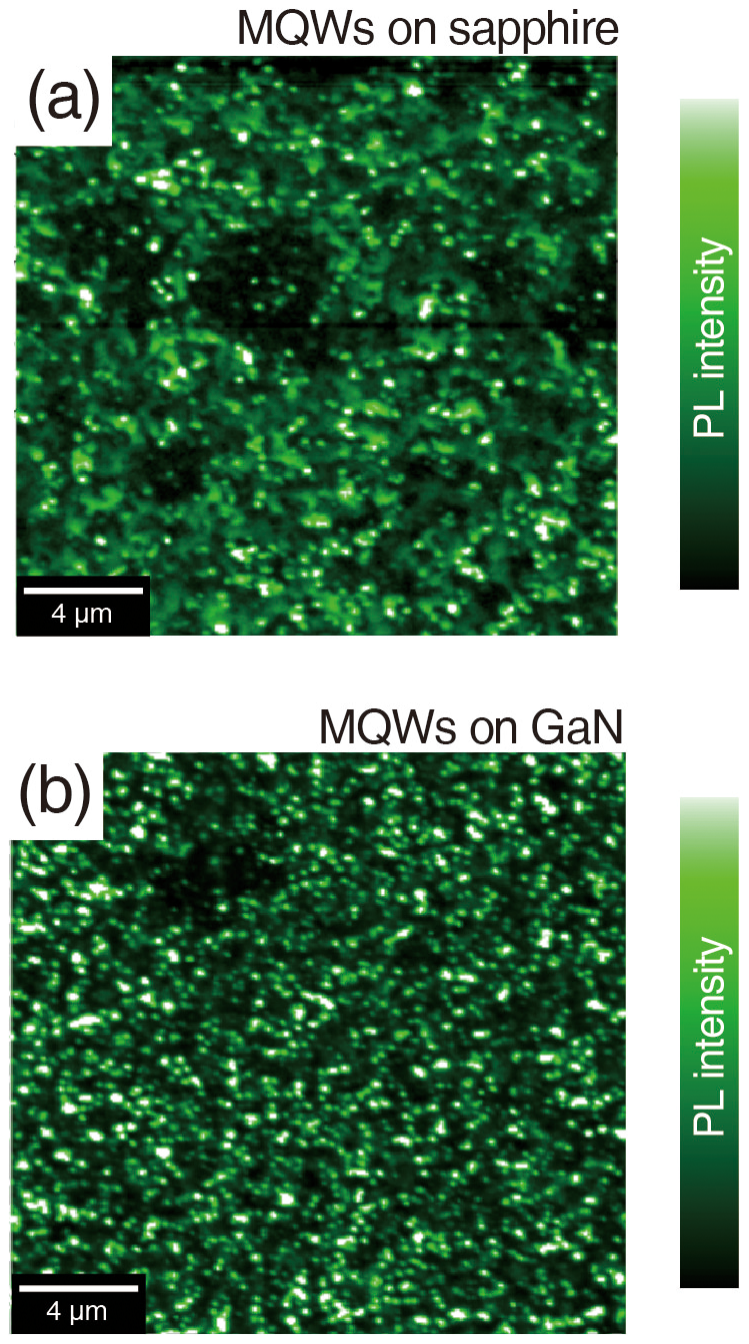
NSOM-PL images of MQWs grown on (a) sapphire and (b) GaN. Both shape and distribution of the luminescent clusters are different depending on the substrate on which the MQWs were grown.

To further investigate the PL characteristics versus the crystalline quality of In-rich InGaN QWs, NSOM was employed, which was performed in contact mode with a cantilever tip with a 100 nm aperture. The 375 nm laser diode was used as an excitation source to only excite the InGaN QWs. The PL emission was collected by an objective lens underneath the sample and detected by a photomultiplier tube at room temperature. Figs. 3(a) and (b) are NSOM-PL images of the MQWs sample grown on sapphire and GaN, respectively. The scan size is 20 μm × 20 μm for both images. As represented by the scale bar of the PL intensity, the white region corresponds to PL intensities. The NSOM-PL image of the MQWs grown on sapphire comprises a blurred luminescent area and luminescent spots, while that of the MQWs grown on GaN is only composed of luminescent spots, as depicted by Figs. 3(a) and (b), respectively. Moreover, the density and uniformity of the luminescent spots are much higher for the MQWs grown on GaN than for those grown on sapphire. (see supplementary information, Figure S1) The luminescent clusters are intimately related to carrier localization as the probability of radiative recombination increases with carrier density^47^,^48^. Consequently, the distribution of luminescent clusters, which is revealed in the NSOM-PL images, reflects that the carrier localization is strongly correlated with the crystalline quality of the sample. (see supplementary information, Figure S2)

**Figure 4:**
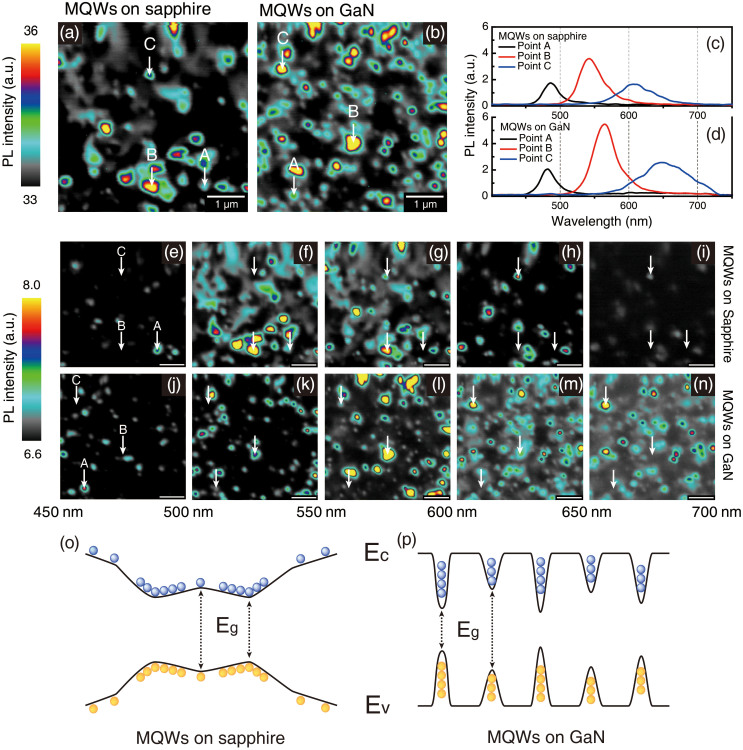
Panchromatic NSOM-PL images of MQWs grown on (a) sapphire and (b) GaN. Only spot-like luminescent clusters are observed in MQWs grown on GaN. Local PL spectra measured at points A, B, and C for MQWs grown on (c) sapphire and (d) GaN. A longer PL wavelength range and higher PL intensity is observed for MQWs placed on GaN. NSOM-PL images of MQWs grown on (e)–(i) sapphire and on (j)–(n) GaN using a 50 nm band-pass filter. The band-pass wavelength ranges for image filtering are given by (e, j) 450 nm–500 nm, (f, k) 500 nm–550 nm, (g, l) 550 nm–600 nm, (h, m) 600 nm–650 nm, and (i, n) 650 nm–700 nm. The emission of MQWs grown on GaN occurs at a higher PL wavelength when compared to MQWs grown on sapphire. Sketch of the energy band diagram for MQWs grown on (o) sapphire and (p) GaN. The deep and narrow potential valleys for MQWs on GaN can be explained by the NSOM results.

To confirm that the carrier localization phenomena are related to the luminescent clusters as shown in the NSOM images, spatially resolved spectroscopic NSOM measurements were carried out. The PL emission was collected by an objective lens and sent to the monochromator via a multimode optical fiber. Through synchronization of the spectroscopic signal acquisition with the NSOM scanner, we obtained the spectroscopic PL mapping images, each consisting of 256 × 256 pixels. Panchromatic NSOM-PL images of MQWs on sapphire and GaN are shown in Figs. 4(a) and (b), respectively. The scan size is 5 μm × 5 μm for both samples. A combination of a blurred luminescent area and luminescent spots is observed for MQWs located on sapphire, whereas only luminescent spots are revealed for MQWs grown on GaN. These results are consistent with the previous NSOM results. From the complete scanned area, we selected three different points for plotting the local PL spectra, which are representative for the entire scan area. Points A and C represent the shortest and longest PL emission, whereas point B corresponds to the region with the highest PL emission intensity. Figs. 4(c) and (d) show the local PL spectra corresponding to the characteristic points A, B, and C, which are measured for MQWs grown on sapphire and GaN, respectively. The peak positions of the PL spectra corresponding to point B are well matched to the macro-PL spectra shown in Figs. 2(a) and (b). The spatially resolved PL spectral range is wider for the MQWs grown on GaN than for those grown on sapphire. More precisely, for wavelength above 650 nm, the PL intensity of the MQWs grown on GaN is higher than that of MQWs grown on sapphire. (see supplementary information, Figure S3) To simplify the spectroscopic analysis of the NSOM results, wavelength-dependent NSOM-PL images with spectral intervals of 50 nm are shown in Figs. 4(e)–(n). The scale bar is 1 μm for all images, while the white arrows indicate the corresponding position of points A, B, and C as depicted in Figs. 4(c) and (d). The full spectral range covered by the NSOM images goes from 450 nm to 700 nm. Figs. 4(e)–(i) are wavelength-dependent NSOM images for MQWs grown on sapphire. The obtained PL intensity is the highest for wavelengths ranging from 500 nm to 550 nm, and is dramatically reduced for longer wavelengths. In the dominant wavelength range, a blurred luminescent area and luminescent spots appear simultaneously, which is in agreement with previous results (Fig. 3). However, only luminescent spots with weak intensity are observed at shorter and longer wavelengths. Wavelength-dependent NSOM-PL images of MQWs on GaN are shown in Figs. 4(j)–(n). The highest PL intensity was observed for wavelengths ranging from 550 nm to 600 nm, and this quantity did not decrease much as the center wavelength was further increased as shown in Figs. 4(m) and (n). Longer PL emission wavelengths hint at radiative recombination in a lower band gap. Furthermore, for MQWs grown on GaN, only spot-like luminescent clusters were observed along the entire analysed wavelength range. These results support that InGaN/GaN MQWs with high crystalline quality contain small and deep potential valleys. Based on the experimental results, we could schematically describe the energy band gap of In-rich InGaN QWs grown on sapphire and on GaN as shown in Figs. 4(o) and (p), respectively. In the case of InGaN QWs grown on sapphire, wide and shallow potential valleys can also occur in PL spectra with low PL intensities and at lower wavelengths. On the other hand, in the case of the MQWs grown on GaN, narrow and deep potential valleys can explain the longer wavelength range of the PL spectrum that exhibits the highest intensity. As a partial summary, via spectroscopic analysis, we also confirmed that carrier localization of In-rich InGaN/GaN MQWs is strongly influenced by the crystalline quality.

**Figure 5:**
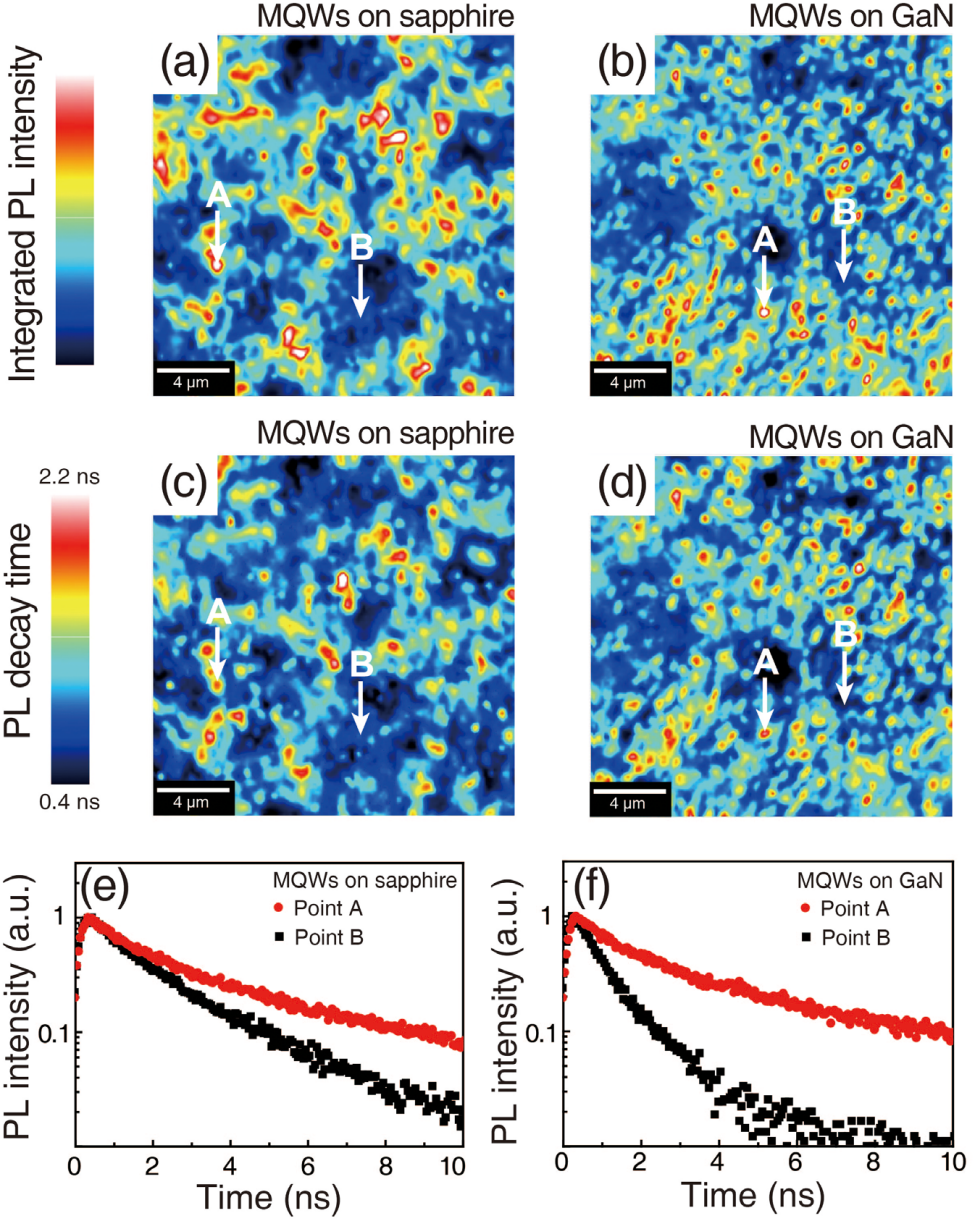
Confocal PL mapping images of MQWs grown on (a) sapphire and (b) GaN. The highest density of luminescent clusters with smaller sizes is observed for MQWs grown on GaN. Confocal TRPL mapping images of MQWs grown on (c) sapphire and (d) GaN. Only for MQWs grown on GaN, the intense PL intensity regions match regions of longer PL decay times well. Local PL decay curves measured at points A and B for MQWs grown on (e) sapphire and (f) GaN, as marked in the above confocal mapping images. The PL decay time difference between points A and B is higher for MQWs grown on GaN.

To further confirm the correlation between luminescent clusters and carrier localization phenomena, an investigation of the carrier dynamics was conducted using confocal microscopy combined with the TCSPC technique. A 405 nm pulsed laser was used as an excitation source and the spatial resolution of the confocal image was around 200 nm. Figs. 5(a) and (b) are the PL intensity mapping images of MQWs grown on sapphire and GaN, respectively. The scan area is 20 μm × 20 μm, and each image consists of 256 × 256 pixels. Large luminescent clusters with low density are observed for MQWs grown on sapphire, while small luminescent clusters with high density are found for MQWs grown on GaN. Note that the blurred luminescent area and luminescent spots cannot be distinguished in the confocal PL images due to the lower spatial resolution of this method when compared to NSOM. Mapping images of the PL decay time are presented in figs. 5(c) and (d) for MQWs grown on sapphire and GaN, respectively. The area corresponding to a longer PL decay time indicates an In-rich InGaN region, which satisfies the condition of carrier localization^49^,^50^. In the case of the MQWs grown on sapphire, higher PL intensity regions do not completely match with regions characterized by longer decay times. In other words, the trend observed for the PL intensity in the mapping image is different from the one observed for the PL decay time. This means that intense PL regions do not only coincide with In-rich InGaN regions. On the contrary, for MQWs grown on GaN, the regions of higher PL intensity match well those corresponding to a longer PL decay time. This behaviour reflects that higher PL intensity regions are In-rich InGaN regions. Fig. 5(e) and (f) are local PL decay curves measured at points A and B as marked in the confocal mapping images, respectively. All curves are normalized to facilitate their mutual comparison. Points A and B are representative positions for high and low PL intensities, respectively. A small difference in the PL decay time between points A and B was observed for MQWs grown on sapphire. Meanwhile, a large difference in PL decay time was detected from both points for MQWs grown on GaN. These results include that luminescent clusters of MQWs grown on sapphire are not only governed by the amount of In content in the InGaN QWs, which is not the case for MQWs grown on GaN. To study the carrier dynamic in details, the PL decay curves have been fitted by using the stretched exponential function:
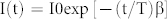
where β is the stretching parameter and I(t) is the time-dependent PL intensity^51^. The stretching parameter β was concerned by localized states in the QW, and is closely related to the crystalline quality^52^. All parameters extracted from the fitting process are presented in Table 1. The parameters extracted from point A are almost identical for both samples. However, for the point B entirely different parameters were found for each sample. Notably, a much higher stretching parameter β was observed for MQWs grown on sapphire. This higher value of β implies that the lower PL intensity of MQWs on sapphire substrates was caused by crystal defects. Moreover, the fast and slow PL decay times corresponding to point B for the sapphire sample are about 70% and 65% longer than those measured for the GaN sample. Longer PL decay times could be explained by carrier trapping, which is caused by defect states in forbidden bands. These observations also support that the lower PL intensity of MQWs grown on sapphire is mainly caused by the lower crystalline quality.

## Discussion

Carrier localization of In-rich InGaN/GaN MQWs was investigated via spatially resolved optical characterization using NSOM and confocal TRPL. To assess the correlation between carrier localization and crystal quality, sapphire and GaN have been used as substrates for the growth of InGaN/GaN MQWs. We found a strong correlation between the carrier localization and the crystal quality based on temperature dependent PL results. The distribution of luminescent clusters on both samples was shown to be quite different as revealed by the NSOM analysis. The clear relation between the luminescent clusters and the longer PL decay time regions strongly support the claim that carrier localization is strongly affected by the crystal quality. Accurate understanding of the optical characteristics of In-rich InGaN/GaN MQWs is the way to overcome the “green gap” issue, which would ultimately lead to pure white LEDs.

## Methods

### Topographical characterization

The topography of the sample surfaces was probed by commercial atomic force microscopy (AFM) in contact mode (Anasys Instruments).

### Optical characterization

For the macro-PL measurements, a 30 cm monochromator (SP2300, Princeton Instrument) was employed to disperse the collected light, a thermoelectrically cooled charge-coupled device (CCD) was used to detect the optical signal (PIXIS100, Princeton Instrument), and furthermore, a He–Cd continuous wave laser operating at 325 nm (IK3501R-G, KIMMON)with a power of 50 mW was used as an excitation source. The temperature was controlled between approximately 10 K and 300 K using a closed-cycle cryostat (ST-100, JANIS).

### Near-field scanning optical microscopy (NSOM)

NSOM was performed by a WITec Alpha SNOM, using a silicon cantilever tip with a ~100 nm aperture. A solid-state laser operated at 375 nm was used as the excitation source. The PL emitted from local sample areas was collected by a microscope objective in transmission mode. The light collected from the objective was sent to a monochromator through a multimode optical fiber and was detected using a thermoelectrically cooled CCD detector.

### Time-resolved Photoluminescence (TRPL)

For analysis of TRPL, a multifunctional confocal microscopy including a time-correlated single photon counting (TCSPC) system was employed (NTEGRA, NT-MDT). For the TRPL excitation source, a 405 nm pulsed laser with a repetition rate of 80 MHz and a pulse width of 80 ps was used. A high-speed PMT detector (PMC-100, Photonic Solutions) was applied for TCSPC photon counting.

## Author Contributions

H.J. carried out experiment measurements, data analysis and manuscript preparation. The temperature-dependent PL spectra conducted by H.J.J., H.M.O. carried out measurement of AFM topographic images. Crystal growth of both samples was performed by C.-H.H. and E.-K.S., G.L. and M.S.J. contributed to experiment planning, data analysis and manuscript preparation.

## Supplementary Material

Supplementary Information Supplementary information

## Figures and Tables

**Table 1:** TRPL parameters extracted from PL decay curves of bright and dark regions on the both of sapphire and GaN

	A_1_	A_2_	τ_1_ (ns)	τ_2_ (ns)	β
Bright area (A) on sapphire	0.554	0.619	0.892	3.470	0.685 ± 0.005
Dark area (B) on sapphire	0.348	0.926	3.508	1.161	0.748 ± 0.005
Bright area (A) on GaN	0.607	0.589	0.815	3.542	0.661 ± 0.006
Dark area (B) on GaN	0.160	1.325	2.127	0.684	0.598 ± 0.012
