# Supplementary material for: Carrier localization in In-rich InGaN/GaN multiple quantum wells for green light-emitting diodes
Source: Sci Rep. 2015 Mar 20;5:9373. doi: 10.1038/srep09373 (PMC4366764; doi:10.1038/srep09373)
Supplement: Supplementary Information [file srep09373-s1.doc]

Supplementary Information

Carrier localization in In-rich InGaN/GaN multiple quantum wells for green light-emitting diodes

Hyun Jeong^1,2^, Hyeon Jun Jeong^2,3^, Hye Min Oh^2,3^, Chang-Hee Hong^4^, Eun-Kyung Suh^4*^, Gilles Lerondel^1,3,*^, Mun Seok Jeong^2,3,*^

*Correspondence to [mjeong@skku.edu, lerondel@utt.fr, eksuh@jbnu.ac.kr]

^1^Laboratoire de Nanotechnologie et d’Instrumentation Optique, Institut Charles Delaunay, CNRS-UMR 6281, Université de Technologie de Troyes, BP 2060, 10010 Troyes, France, ^2^Center for Integrated Nanostructure Physics (CINAP), Institute for Basic Science (IBS), Sungkyunkwan University, Suwon 440-746, Republic of Korea, ^3^Department of Energy Science, Sungkyunkwan University, Suwon 440-746, Republic of Korea, ^4^School of Semiconductor and Chemical Engineering, Chonbuk National University, Jeonju 561-756, Republic of Korea

**S1. Fourier transform of NSOM images**

NSOM-PL images of InGaN/GaN MQWS on sapphire and GaN were converted by Fourier transform for estimating disorder in terms of size of luminescent clusters. Fig. S1 (a) and (b) are transformed images for MQWs on sapphire and GaN, respectively. Original images are NSOM-PL images shown in Fig. 3 in the main text. In MQWs on sapphire, relatively higher back ground intensity and lower center intensity are observed compared to MQWs on GaN. It indicates that size of luminescent clusters revealed in NSOM-PL images are more uniform in the MQWs on GaN compared to MQWs on sapphire. This result is well matched to spectroscopic analysis interpreted in Fig. 4 in the main text.


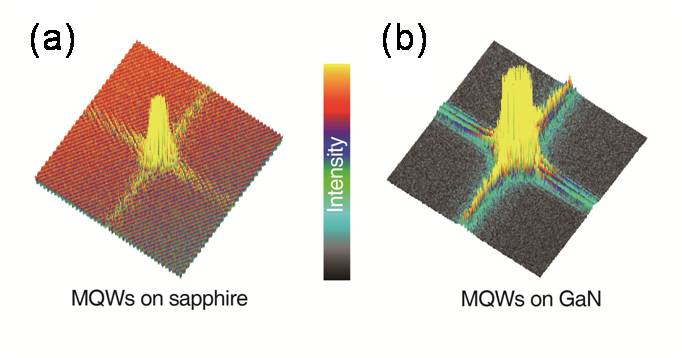


Figure S1. NSOM images converted by Fourier transform for MQWs on (a) sapphire and (b) GaN.

**S2. Intensity statics of NSOM-PL images**

For analysis of intensity statics, histogram of PL intensities is extracted from the NSOM-PL images shown in Fig. 3. Fig. S2 is integrated PL intensity versus measured area which means number of pixel in the NSOM images. Black and red bars indicate MQWs on sapphire and GaN, respectively. As shown in Fig. 2S, in MQWs on GaN, higher number of pixel reveled in higher PL intensity region. Since x-axis is integrated PL intensity which includes whole wavelength, this means total brightness of MQWs on GaN is definitely higher than MQWs on sapphire.


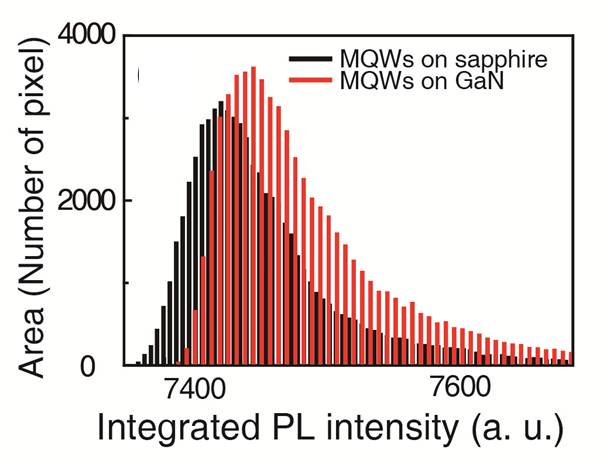


Figure S2. PL intensity histogram of MQWs on sapphire and GaN extracted from NSOM-PL images.

**S3. Spectroscopic statics of NSOM-PL images**

For spectroscopic statics, average PL spectra for MQWs on sapphire and GaN are extracted from NSOM-PL images. Fig. S3 is average PL spectra for both samples. Black and red solid lines are PL spectra for MQWs on sapphire and GaN, respectively. As shown in the average PL spectra, MQWs on GaN has more wide spectral range than MQWS on sapphire. Especially, in the lower energy region below ~2.2 eV, much higher PL intensity was observed in the MQWs on GaN compared to MQWs on sapphire. This result is in correspondence with spectroscopic analysis of NSOM-PL images as revealed in Fig. 4.


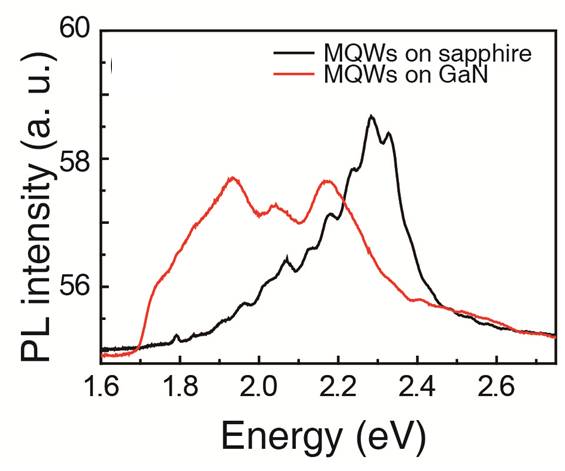


Figure S3. Average PL spectra for MQWs on sapphire and GaN extracted from NSOM-PL images.
